# Identification of different cDNA clones of the Apomixis-specific gene-1 homolog involved in aposporous embryo sac formation in guineagrass (*Panicum maximum* Jacq.)

**DOI:** 10.5511/plantbiotechnology.26.0428a

**Published:** 2026-06-25

**Authors:** Lanzhuang Chen, Liming Guan, Taiji Adachi

**Affiliations:** 1 Graduated School of Horticultural and Food Science, Minami Kyushu University, 3764-1 Tateno-cho, Miyakonojo, Miyazaki 885-0035, Japan; 2 Faculty of Environmental and Horticultural Science, Minami Kyushu University, 3764-1 Tateno-cho, Miyakonojo, Miyazaki 885-0035, Japan; 3 Faculty of Education and Culture, University of Miyazaki, Miyazaki 889-2192, Japan; 4 Faculty of Agriculture, University of Miyazaki, 1-1 Gakuen Kibanadai, Miyazaki 889-2192, Japan

**Keywords:** apomixis, aposporous embryo sac formation, different cDNA clones of the Apomixis-specific gene 1 homolog (*ASG-1H*), guineagrass (*Panicum maximum* Jacq.), in situ hybridization

## Abstract

Apomixis is the reproduction mode in which only the mother’s genes are transmitted from generation to generation. If this trait can be put to practical use, it is expected to be a truly epoch-making breeding method, as seed production costs can be greatly reduced by fixing F_1_ hybrids. This study aimed to isolate the aposporous apomixis gene and analyze how the gene(s) will be expressed from/in the aposporous guineagrass (*Panicum maximum* Jacq.). A new classification method using the ovary length as an index was developed to sample different developmental stages of ovaries and buds in obligate sexual plants and apomicts. A cDNA library was derived from the ovaries of aposporous accession N68/96-8-o-11 staged at the appearance of aposporous initial cells (AICs) to isolate AIC stage-specific genes. Using differential screening, four AIC stage-specific cDNA clones obtained from ten thousand of plaques by Northern blot hybridization showed the same start codon and sequences, ranging in lengths from 577 to 1182 bp. The characteristics and their homologies of the four cDNA clones are similar to Apomixis-specific gene-1 (*ASG-1*), indicating that they are the different cDNA clones of Apomixis-specific gene-1 homolog (*ASG-1H*). In situ expression analysis detected signals without distinguishing between *ASG-1* mRNA and the *ASG-1H* on gene expression specifically in AIC, AIC-derived embryo sacs, and root tips and shoot apical meristems of aposporous accession. The finding and identification of *ASG-1H* expressed at the times of AIC appearance and AIC-derived embryo sac formation, may represent an initial step towards isolating an apospory gene.

## Introduction

Apomixis, the asexual formation of seeds in plants, leads to populations that are genetically uniform maternal clones (Ravi et al. 2008), in which apomictic plants form true seeds originating clonal offspring genetically identical to the mother (Nogler 1984). Because apomixis provides a method to clone plants through seeds and conserves heterozygosity, its use in breeding programs is expected to simplify the development of hybrid cultivars and production of commercial hybrid seeds (Asker 1979; Nogler 1984). The transfer of apomixis to crop plants holds great promise for the fixation of hybrid vigor, allowing for the propagation of hybrids over successive generations (Koltunow et al. 1995). Genetic engineering may be a more rapid approach to reaching this goal. However, to make genetic engineering approaches feasible, we must first attain a better understanding of the molecular mechanism controlling apomixis (Guerin et al. 2000; Koltunow 1993; León-Martínez and Vielle-Calzada 2019).

Three types of apomixis have been identified: 1) a sporophytic form of adventitious embryony and the gametophytic forms of 2) diplospory and 3) apospory (Asker and Jerling 1992). Gametophytic apomixis involves 2 steps—embryo sac formation and parthenogenesis (Barcaccia and Albertini 2013; Hand and Koltunow 2014).

In aposporous apomixis research, cytological and molecular studies on potentially causative apomixis genes have been conducted in some monocotyledon crops. In *Pennisetum squamulatum*, apomixis is transmitted by a physically large, hemizygous, nonrecombining chromosomal region known as the apospory-specific genome region (ASGR) (Akiyama et al. 2005; Ozias-Akins et al. 1998). Conner et al. (2015) transformed a single gene (BABY BOOM-LIKE) contained on ASGR into a sexual, recombining region and obtained unfertilized seeds through parthenogenesis, the second step of the apomixis process. Until now, a confirmed apomixis gene cloned from the aposporous grass of *Pennisetum squamulatum* has been reported and is involved in parthenogenesis (Conner et al. 2015). Recently, Khanday et al. (2019), has reported that zygotic expression of BBM1 initially specific to the male allele but is subsequently biparental, and this is consistent with its observed auto-activation, and ectopic expression of BBM1 in the egg cell is sufficient for parthenogenesis, which indicates that a single wild-type gene can bypass the fertilization checkpoint in the female gamete. However, the first step, that is, the molecular mechanism of embryo sac formation, is yet unknown.

In *Paspalum notatum*, aposporous initial cells (AICs), which is originated from nucellar tissue around the megaspore, appeared with a sharp knife, and inserted the space that the degenerated megaspore left (Chen et al. 2000), and multiple seed-forming embryos were formed in the same polyembryonic ovule (Chen, Guan et al. 2001).

In *Panicum maximum*, it exists as a varietally apomictic mass, i.e., obligate sexual, facultative and obligate apomixis (Asker and Jerling 1992; Koltunow 1993). An effective and rapid method for estimating the degrees of sexuality or apomixis is, therefore, needed to be developed for their utilization in various plant breeding programs. Up to now, to estimate the degree of sexuality, two methods, i.e., embryo sac analysis and progeny test were often combined and used by many workers (Hanna et al. 1973; Nakagawa 1990; Nakajima and Mochizuki 1983; Savidan 1975; Savidan and Pernès 1982; Smith 1972; Warmke 1954). However, it remains unknown in facultative apomicts which embryo sac will become the seed-forming one, as two types of sexual and apomictic embryo sacs coexist in a same ovule. Chen and Kozono (1994b) have reported that the degrees of sexuality or apomixis in facultative apomicts, can be estimated based the proportion of sexual and apomictic embryo sacs in the micropylar end by means of embryo sac analysis in present generation, even without progeny test, using Nomarski differential interference-contrast (DIC) microscopy. And then, they have clarified that, when comparing the both of reproduction processes in sexual and apomict, aposporous embryo sac initial cell (AIC) derived from the unreduced nucellar cell, appeared while the functional megaspore was usually still existing without clear membranes at chalazal end using DIC (Chen and Kozono 1994a), and while the mother megaspore cell was degenerated using transmission electron microscopy (TEM) (Guan et al. 2006, 2007). According to the index of a novel classification method based on the ovary to sample different developmental stages of embryo sac formation in sexual plants and apomicts, Apomixis-specific gene-1 (*ASG-1*) cDNA was isolated during the stage in which the AICs appear using the differential screening method (DS) (Chen et al. 1999). This cDNA was expressed only during the AIC stage. Comprising 1,177 base pairs, *ASG-1* cDNA encodes a 305-amino acid protein. The homology of *ASG*-1 is similar to that of RD22, a seed-specific, and drought-induced gene of *Arabidopsis thaliana* (Yamaguchi-Shinozaki and Shinozaki 1993), and USP, an unknown seed protein precursor of *Vicia faba* (Bäumlein et al. 1991). In situ hybridization indicated that *ASG-1* is expressed during the AIC stage in aposporous plants but not in any stage in sexual plants. However, strong expression of *ASG-1* was detected in immature pollen grains and young embryos of both reproductive types, suggesting that *ASG-1* may be an allele derived from the obligate sexual wild type (Chen et al. 2005). The possibility that *ASG-1* is a male gamete-specific gene could not be excluded because it was isolated from AIC-staged young buds containing both the anther and ovary (Chen et al. 1999).

In another approach in *P. maximum*, a genetic linkage map of guinea grass (*P. maximum* Jacq.) was generated with nine of the AFLP markers found to be associated with apospory (Ebina et al. 2005). And then, the DNA contents of one diploid strain and three tetraploid cultivars of *P. maximum* were examined by flow cytometry and rDNA loci were physical mapped by fluorescence in situ hybridization (Akiyama et al. 2008). And more, recently, it is reported that when the BABY BOOM1, a member of the AP2 family of transcription factors expressed in sperm cells, was used in egg cells for ectopic expression, the single wild-type gene can bypass the fertilization checkpoint for parthenogenesis sufficiently in rice (*Oryza sativa*) (Khanday et al. 2019). And Underwood et al. (2022), have reported that A *PARTHENOGENESIS* allele from apomictic dandelion can induce egg cell division without fertilization in lettuce. And more, synthetic apomixis can be achieved in an F1 hybrid of rice by inducing MiMe mutations and egg cell expression of BBM1 in a single step, and which produce more than 95% of clonal seeds across multiple generations (Vernet et al. 2022). However, up to now, the real apomixis genes have not been isolated from the true gametophytic apomicts of not only aposporous but also diplosporous plants.

This study focused on investigating the molecular mechanism of AIC appearance, from which the aposporous embryo sac is formed, and was planned to isolate the aposporous apomixis gene and analyze how the gene(s) will be expressed from/in the aposporous *P. maximum* Jacq. We have used a modified classification standard based on the ovary length as an index to allow the sampling of AIC-stage ovaries, but not the young buds containing male gametes using the DS method, and searched for AIC stage-specific genes by Northern and Southern blot hybridization, and sequencing and homology analysis, followed by in situ hybridization of the obtained genes expressed in AIC, AIC-derived embryo sac (ES) formation, and their development of ES, as well as in the vegetative organs in aposporous and sexual accessions.

## Materials and methods

### Plant materials

We used both reproductive types of guinea grass (*P. maximum* Jacq.): a facultative apomict (N68/96-8-o-11) with a degree of apospory of 94% (2n=4x) and an obligate sexual strain (N68/96-8) (Chen and Kozono 1994a). The plants were cultivated in a greenhouse at the farm of Minami Kyushu University, Miyazaki, Japan. Using the ovary length as an index, the young buds were collected using a modified version of the classification method of Chen et al. (1999). Whole ears containing different developmental stages of guinea grass were collected, placed in beakers of water, and brought to the laboratory for ovary collection.

### RNA isolation, cDNA synthesis, and cDNA library construction

We used the Aqua Pure RNA kit (Bio-Rad, Tokyo, Japan) and 1000 ovaries (ca. 5 mg) to extract total RNA from the A2 stage. For young buds of the other stages, a total RNA separator kit (Clontech, Mountain View, CA, USA) was used. Poly(A)^+^ RNAs were purified using the Oligotex-dT30 system (Takara Bio, Shiga, Japan), and double-strand cDNA synthesis and the cDNA library from A2-stage ovaries were constructed using the Uni-ZAPII-cDNA synthesis kit (Stratagene, La Jolla, CA, USA) according to the method described by Chen et al. 1999.

### Differential screening of the A2-stage cDNA library

The AS1-, A2-, and S2-stage cDNAs were used as probes. Plaques (10^5^) from the primary cDNA library, constructed from stage A2 ovaries, were plated (10^3^ plaques per 154 mm dish), transferred to nylon membranes (Hybond-N, Amersham, UK), and differentially screened with ^32^P-labeled cDNAs from all three samples. Plaque hybridization and in vitro excision of the recombinant phage were performed according to the manufacturer’s protocol (Stratagene). Northern blot analysis was conducted according to the method of Chen et al. (1999).

### DNA extraction and Southern blot analysis

Total DNA was extracted from the leaves of both aposporous and sexual accessions of guinea grass according to Honda and Hirai (1990). Southern hybridization was conducted as described by Southern (1975). DNA (5 mg) from both accessions was digested in separate reactions with 2 mg each of the restriction enzymes *Bam* HI, *Bgl* II, *Eco* RV and *Hind* III for 5–6 h at 37°C in 20 ml of H buffer (Takara Shuzo, Japan). Restriction fragments were separated by electrophoresis into 0.6% agarose slab gels containing 1×TAE for 15 h. After denaturation, the DNAs were transferred onto nylon membranes. ^32^P-labeled plasmid DNA, containing a putative aposporous gene (A2-512), was used as a probe, and Southern hybridization was carried out following the manufacturer’s instructions. The hybridized fragments were visualized by autoradiography.

### cDNA sequencing and homology search

Cloned cDNA fragments of A2-132, -133, -231, and -512, cleaved by restriction enzymes, were subcloned into six overlapping plasmids, cloned into pBluescript II SK^−^ (Stratagene), and sequenced according to the method of Chen et al. (1999).

### Synthesis of the probe for in situ hybridization

The apomixis-specific genes of A2-132, A2-133, A2-231, and A2-512 were cloned in this study. Because these clones have the same sequences with different lengths, the clone with the longest sequence (A2-132 cDNA) was chosen for use as the probe. Briefly, the plasmid (UniZAP, Stratagene) A2-132 cDNA was digested with *EcoR* I and *Xho* I for linearization. For antisense RNA probe synthesis, the latter linearized cDNA was ligated with the T3 promoter, followed by the addition of the DIG-RNA labeling mixture (Behringer Mannheim), RNase inhibitor (Takara Bio), T3 RNA polymerase (Stratagene), and transcription buffer. For sense probe synthesis, the former cDNA was ligated with T7, and the other steps were similar to those of T3. For details, the probe synthesis was performed as described previously (Chen et al. 2005).

### Staging of the ovary, berry, and other organs selected for in situ hybridization

The floral staging of both sexual and aposporous accessions of guinea grass was carried out as described by Chen et al. (2005). For stage classification, the buds, young flowers at anthesis, and young berries were collected and categorized according to the ovary length and days after anthesis (DAA). The ovaries were isolated from aposporous plants at four developmental stages representing (1) the completion of megasporogenesis (Stage A1), (2) appearance of AICs (Stage A2-1), (3) appearance of uni-, di-, and tetra-nucleate ESs until anthesis (stage A2-2), and the young berry containing developing embryos and endosperms at 1, 2, and 4 DAA (stage A3). Three stages of ovaries were isolated from sexual plants representing (1) the completion of megasporogenesis (stage S1), (2) onset of regular embryo sac development until anthesis (stage S2), and (3) young berry at 4 DAA (stage S3). For controls, the meristem and fresh roots of both sexual and aposporous guinea grass plants were collected from in vivo living plants cultivated in the field.

### In situ hybridization

Tissues were fixed in 3% paraformaldehyde and 0.25% glutaraldehyde and were embedded in paraffin wax. Paraffin was removed from sections (8 µm thick) with xylene (2 changes), and sections were dehydrated in an ethanol series (100–30%). Protease digestion was carried out by incubation for 30 min at 37°C with proteinase K (5 µg ml^−1^) in 100 mM Tris-HCl (pH 7.5) and 50 mM EDTA. The RNA probe was hydrolyzed to approximately 150 bp by incubation with 0.2 M Na_2_CO_3_ buffer (pH 10.2) and 0.2 M NaHCO_3_ (pH 10.2) for 50 min at 60°C. The slides were hybridized overnight at 50°C with 3 ng µl^−1^ kb^−1^ of digoxigenin-labeled probe in 200 µl of deionized formamide, 5 M NaCl, 100 mM Tris-HCl, 10 mM EDTA, 50× Denhardt’s (Sigma), 50% dextran sulfate, 3M DTT, 100 µg µl^−1^ of tRNA (Boehringer, Mannheim), 10 µg µl^−1^ of poly (A), and sterile water. After hybridization, the slides were treated as described previously (Chen et al. 2005). The sections were dehydrated in an ethanol series, air dried, mounted in Eukitt (ORSA Technologies, Sierra Vista, AZ, USA) and photographed using DIC on a Leica DMI6000B microscope (Wetzlar, Germany).

## Results

### Improvement of ovary collection from young buds

**Figure 1:**
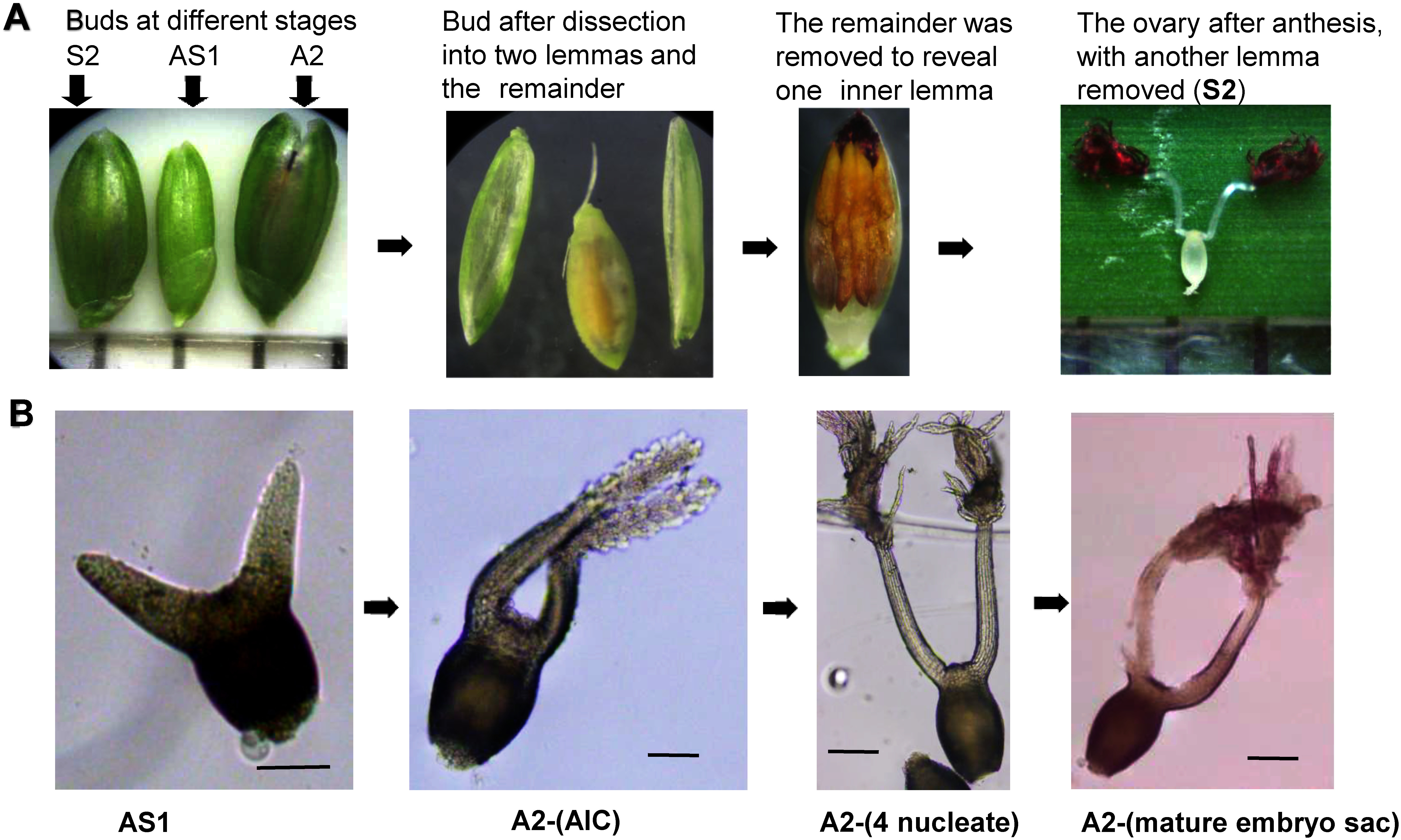
Figure 1. Chart for sampling and collecting ovaries from differently developmental stages of Guinea grass. A. Sampling of young buds according to the ovary length (Chen and Kozono 1994a; Chen et al. 1999) and the process of ovary dissection for total RNA extraction in sexual and apomictic guinea grass. AS1, stage of one mature megaspore; A2, stage of aposporous embryo sac initial cell (AIC) appearance to mature AIC-derived embryo sac; S2, stage of one mature megaspore to mature sexual embryo sac. B. Pistils of different morphology dissected from different developmental stages of apomictic guinea grass. The pistil in AS1 stage shows the appearance of styles (upper) and ovary (lower). Bar=50 µm; A2-(AIC) with stigma showing shorter villi with white color (upper), elongating styles (middle) and ovary (lower). Bar=70 µm; A2-(4-nucleate) with stigma showing longer villi with grey color (upper), fully elongated style (middle) and ovary (lower). Bar=100 µm; A2-(matured embryo sac) with matured and pink colored stigma (upper), style (middle), and ovary (lower). Bar=150 µm.

The young buds were divided into different stages based on the classification scheme using the ovary length as an index (Chen and Kozono 1994a): stage 1, the stage of megasporogenesis; because there is one mature megaspore formation in this stage either in apoamphimictic stage 1 (A1) or in sexual stage 1 (S1) (Chen and Kozono 1994a), we named stage 1 as stage AS1 stage and used A1 of aposporous accession as stage AS1 in this study, similar to that reported previously (Chen et al. 1999); stage 2 (A2), apoamphimictic stage; stage 2 (S2), sexual phase (Figure 1A). The buds of stage A2 were dissected under a microscope; the ovaries were removed and quickly placed into beakers containing LN2 and then were placed on dry ice until extraction. Approximately 1,000 ovaries were collected from A2-stage buds (Figure 1B).

### Identification of cDNA clones from different developmental stages

**Figure 2:**
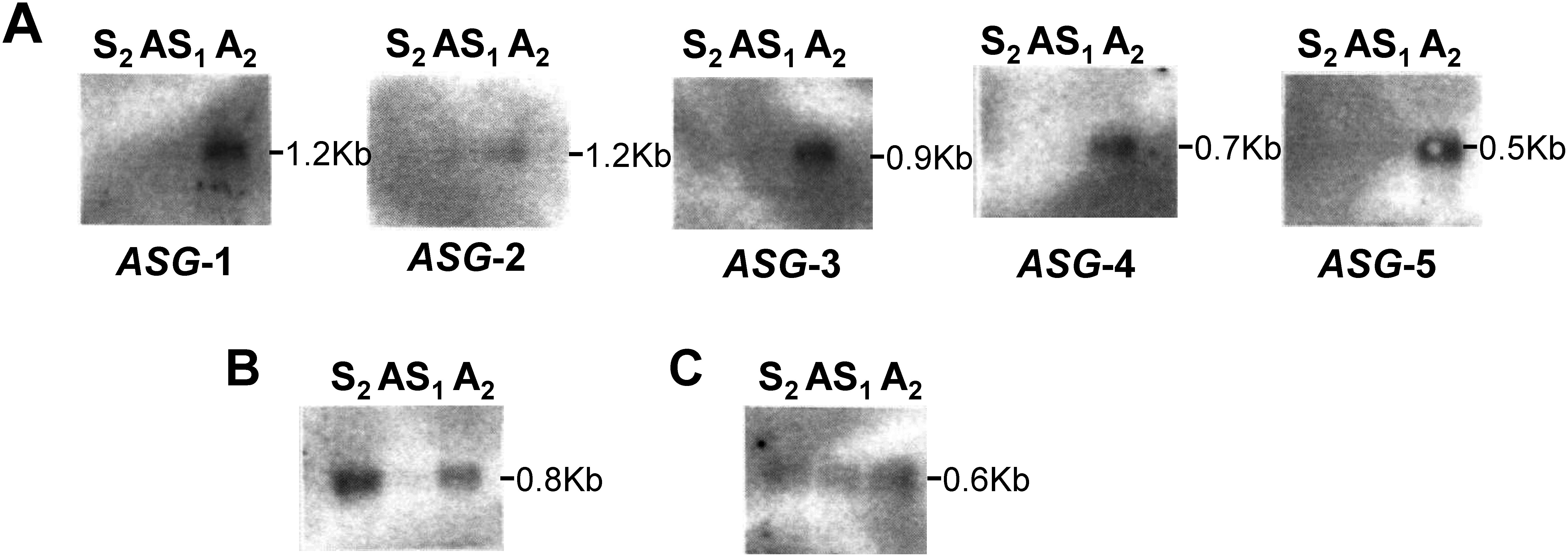
Figure 2. (A) Northern blotting of cDNAs isolated from stage A2 ovaries of facultative apomictic guinea grass. *ASG-1* is derived from A2-134 (Chen et al. 1999) and is used here as a control. *ASG-2*, *ASG-3*, *ASG-4*, and *ASG-5* are from A2-231, A2-512, A2-133, and A2-132, respectively. At this stage (A2), AICs appear and AIC-derived embryo sacs are formed in apomictic, but not in sexual genotypes. The hybridization signals correspond to this stage and are not visible during megaspore (AS1) or sexual embryo sac formation (S2). (B) P332 is a cDNA clone found in both genotypes at stages A2 and S2, respectively and is used here as a control for clone specificity. (C) P232 is a cDNA clone found in both genotypes at stages AS1, A2, and S2 and is used here as a control for clone specificity.

Northern blot hybridization showed that A2-specific clones included A2-512, A2-231, A2-133, and A2-132 (Figure 2A); A2- and S2-specific clones included p122 (Figure 2B); and clones present in all stages included p232 (Figure 2C). The largest clone was A2-231, approximately 1.2 kbp; the other three new clones were smaller than 1.0 kbp.

### Identification of two independent fragments by southern hybridization

**Figure 3:**
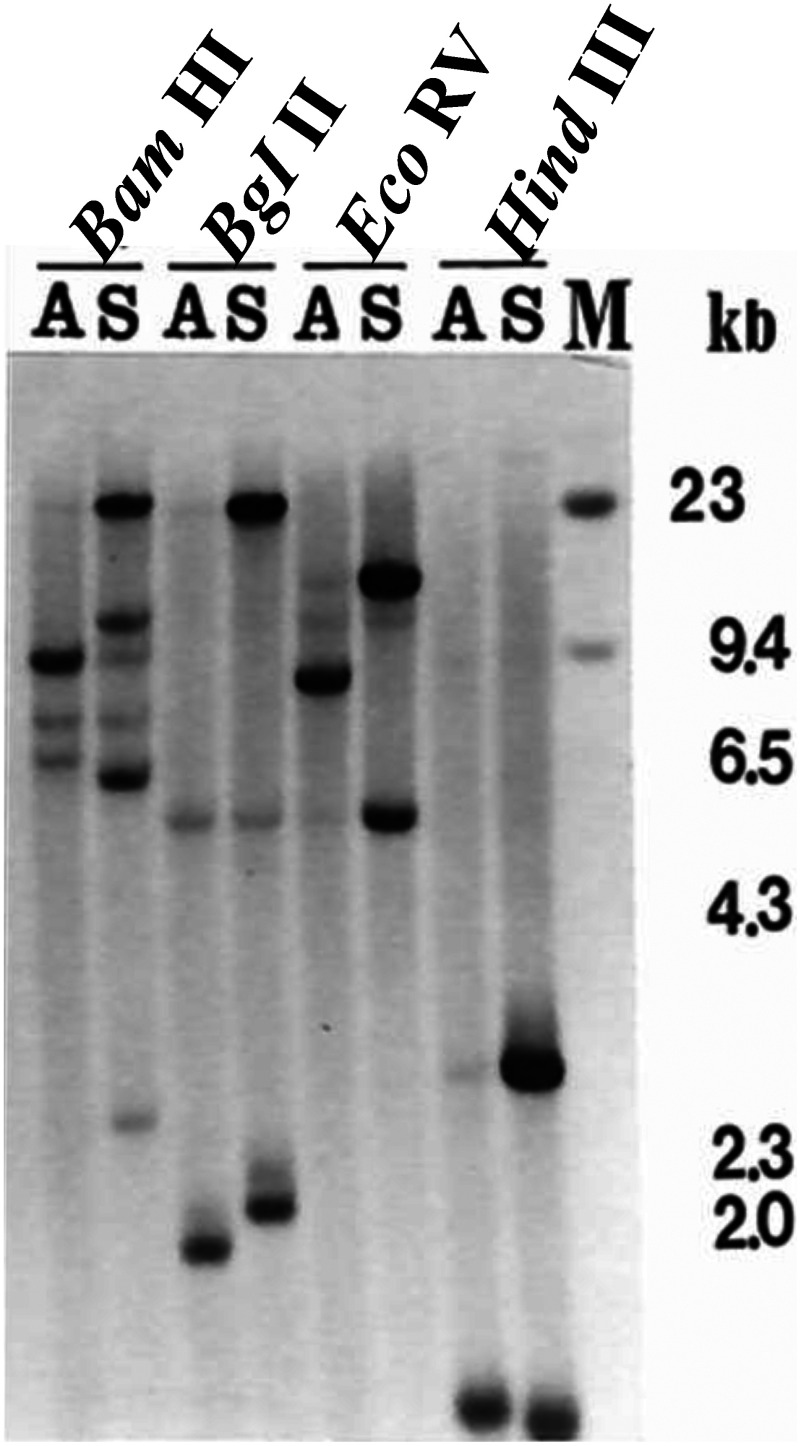
Figure 3. Southern blotting of A2-512 cDNA with total DNA isolated from the leaves of both the sexual (S) and apomictic genotype (A), after digestion with restriction enzymes *Bam* HI, *Bgl* II, *Eco* RV, and *Hind* III. The respective gene or a related member of the same family seems to be present in both genotypes. M=λ DNA/*Hind* III Marker.

To confirm the presence of the A2-512 gene in native DNA, we hybridized A2-512 cDNA with total DNAs isolated from the leaves of both the aposporous (N68/96-8-o-11) and sexual (N68/96-8) accessions. Southern hybridization revealed that (1) the patterns differed between the two accessions for all enzymes used and (2) the number and size of bands, as well as the intensity of most signals, were greater in the sexual accession than in the apomict (Figure 3).

### Identification of four independent A2-specific cDNA clones

**Figure 4:**
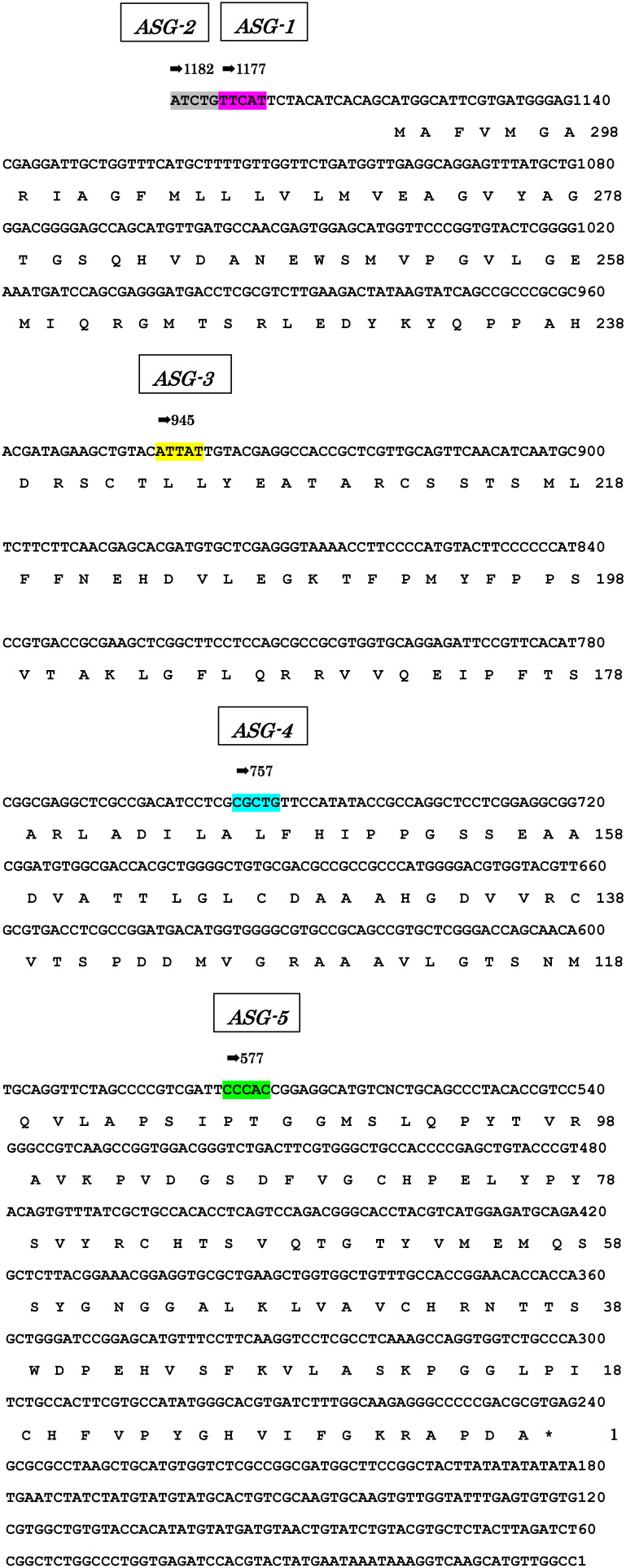
Figure 4. The sizes and structures of the *ASG-1H*’s sequences isolated from facultatively aposporous apomictic guinea grass (N68/96-8-o-11). Their complete nucleotide sequences and deduced amino acids for the major reading frame are shown. The arrows and the colors indicate the numbers and the ends of the nucleotide and amino acid positions of *ASG-1*, *ASG-2*, *ASG-3*, *ASG-4* and *ASG-5* marked in purple, grey, yellow, blue and green lights, respectively. The star (*) indicates the stop codon.

Because the collected clones differed in the length of the DNA fragment from *ASG-1* (A2-134) in Northern blot hybridization, we sequenced the four new cDNA clones. The lengths were as follows: A2-231, 1,182 bp (the longest sequence of the four new clones); A2-512, 945 bp; A2-133, 757 bp; and A2-132, 577 bp. The cDNA clone of A2-231 encodes a protein of 305 amino acids, a 5’-flanking region of 23 bp, and a 3’-flanking region of 244 bp (Figure 4). The other three new clones encode just a part of the 305-amino-acid protein and a 3’-flanking region of 244 bp (Table 1). A surprising result was that the four new cDNA clones have the same sequence but different lengths. We also found that the four new clones contain the same start codon as *ASG-1* (Chen et al. 1999; GenBank accession number AB000809). We conclude that they are the same gene but with different lengths of cDNA. We designated the clones A2-231, A2-512, A2-133, and A2-132 as *ASG-2*, *ASG-3*, *ASG-4*, and *ASG-5*, respectively.

**Table 1:** Table 1. Nucleic acid and amino acid sequence lengths of the *ASG-1H*.

cDNA	Gene	Nucleic acid	Amino acid	Remarks
A2-134	*ASG*-1	1177	305	(AB000809)
A2-231	*ASG*-2	1182	305	Not registered
A2-512	*ASG*-3	945	233	Not registered
A2-133	*ASG*-4	757	170	Not registered
A2-132	*ASG*-5	577	111	Not registered

### Identification of homologs

We also used the Blast-Version 2.1 PIR database to search the 305 amino acids of *ASG*-*2*. The search for sequence homology (Altschul et al. 1990) unveiled a relationship between *ASG*-2 and RD22, a seed-specific, and drought-induced gene of *Arabidopsis thaliana* (Yamaguchi-Shinozaki and Shinozaki 1993), and USP, an unknown seed protein precursor of *Vicia faba* (Bäumlein et al. 1991). This result was identical to that of *ASG-1* (A2-134). When we searched the *ASG-2* clone for amino acid sequences longer than 305, no homology was found. Detailed data concerning homologies are shown in Table 2.

**Table 2:** Table 2. Similarity of the amino acid sequence between the *ASG-1H* and those mainly registered.

Gene	Species	Protein	% homology	
		Function	Identity	Positivity
*ASG*-1	*Panicum maximum*	apomixis-related	100	100
*ASG*-2	*Panicum maximum*	apomixis-related	100	100
*ASG*-3	*Panicum maximum*	apomixis-related	100	100
*ASG*-4	*Panicum maximum*	apomixis-related	100	100
*ASG*-5	*Panicum maximum*	apomixis-related	100	100
*RD*22	*Arabidopsis thaliana*	seed-specific and drought-induced	31–44	49–61
*USP*	*Vicia faba*	unknown seed protein	24–58	51–70
*ADR*6*P*	*Glycine max*	auxin down-regulated	32–58	48–70
*Polyg*1	*Lycopersicon esculentum*	polygalacturonase 1 beta chain precursor	32–38	48–64

### Unique expressions of ASG-2 in different organs and stages of sexual and apomict

*ASG-2* signals were detected in AIC appearance and AIC-derived embryo sac in the N68/96-8-o-11 apomict, but no signal was detected in the S1 or S2 embryo sac in sexual N68/96-8 plants (Table 3). This result of *ASG-2* signals is the same as that observed for *ASG-1* in a previous report (Chen et al. 2005).

**Table 3:** Table 3. *ASG-2* expression in the vegetative and reproductive organs of obligate sexual and facultative apomictic isolates of Panicum maximum*.

Developmental stage and organ	Signal location in sexual N68/96-8	Signal location in apomictic N68/96-8-o-11
*1) Shoot apical meristems*		
Apomict		No signal
Sexual	No signal	
*Root meristem*		
Apomict		Nucleus of root cell
Sexual	No signal	
*Ovary*		
A-1		No signal
S-1	No signal	
A2-1		AIC
S2(early)	No signal	
*Anther*		
A-1		Immature pollen
S-1	Immature pollen	
A2-1		Weak signal in pollen
*2) Ovary*		
A2-2		AIC-derived embryo sac
S2(late)	No signal	
*Anther*		
S2(early)	Weak signal in pollen	
S2(late)	No signal	
A2-2		No signal
*3) Embryo sac* (es)		
S-2DAA	em and en	
S-4DAA	em and en	
A-2DAA		em and en in 1st es
		e and p in 2nd and 3rd es
A-4DAA		em and en in 1st es
		em and p in 2nd es

*Before 1), at 2), and after 3) anthesis. Definition of stages A-1, A2-1, A2-2, S-1, S-2: see Results and Figure 1. S, sexual; A, apomict; e, egg cell; p, polar; em, embryo; en, endosperm; es, embryo sac; DAA, days after anthesis.

**Figure 5:**
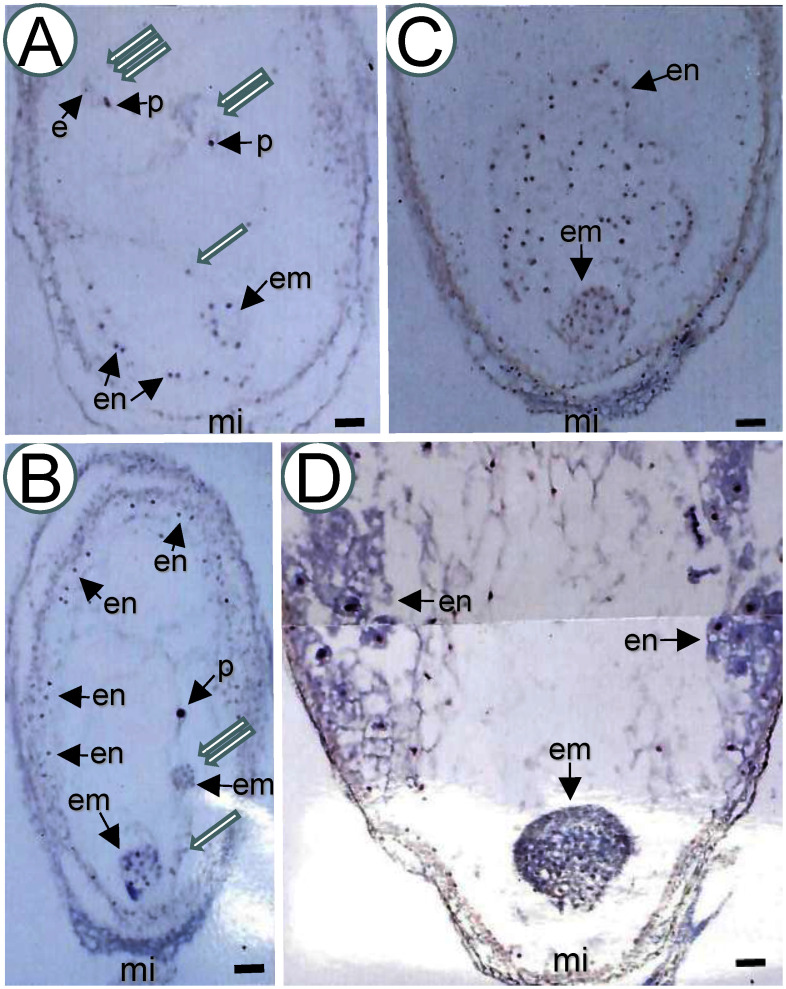
Figure 5. In situ hybridization of *ASG-1* RNA in longitudinal sections of developing ovaries within single and polyembryonic sacs from sexual (C–D) and facultative apomictic (A–B) *Panicum maximum* plants. The expression pattern in sexual and facultative apomictic plants is identical after the anthesis stage. (A) A developing ovary staged 2 days after anthesis (DAA) with three embryo sacs formed: one (single arrow) located at the micropylar end (mi) with an embryo containing eight to sixteen cells (em) and endosperm with free nucleates (en); the second apomictic embryo sac (double arrows) with an observable and undivided polar nucleate (p); and the third (three arrows) containing an undivided egg cell (e) and polar body (p), both located at the chalazal end. (B) A developing ovary staged at 4 DAA with two embryo sacs: one embryo sac (single arrow) located in mi with the embryo containing approximately 64–128 cells (em) and free nucleate endosperm (en); a second embryo sac (double arrows) with 8–32 cells (em) and an undivided polar body (p). (C) A developing ovary with a single embryo sac staged at 2 DAA, containing a well-developed embryo (em) and free nucleate endosperm (en). (D) A developing ovary with a single embryo sac staged at 4 DAA, containing a well-developed, near globular-stage embryo (em) and endosperm (en) (Bars in A, C=50 µm; B, D=100 µm).

To further observe the function of *ASG-2* in embryo and endosperm development after anthesis, the ovaries of the apomict were investigated using the *ASG-2* probe. A strong signal was detected in the ovary containing three embryo sacs at 2 DAA (Figure 5A). Interestingly, the first ES, located at the micropylar end, showed the greatest development, with an 8- to 16-cell embryo and a well-developed endosperm. The other two sacs were located at the chalazal end and contained an undivided egg cell and a polar nucleus. At 4 DAA, the first sac (Figure 5B) had developed both an embryo and endosperm; the other sacs were crowded out to the chalazal end, containing an egg-divided 8- to 16-cell embryo and undivided polar nucleus. The *ASG-2* signal was also observed in the ovaries of the sexual isolate. A strong signal was detected from the ovary containing a well-developed embryo and endosperm at 2 DAA (Figure 5C). At 4 DAA, the ovary contained a heart-like-stage embryo and endosperm with highly concentrated cytoplasm, in which *ASG-2* signals were detected (Figure 5D).

**Figure 6:**
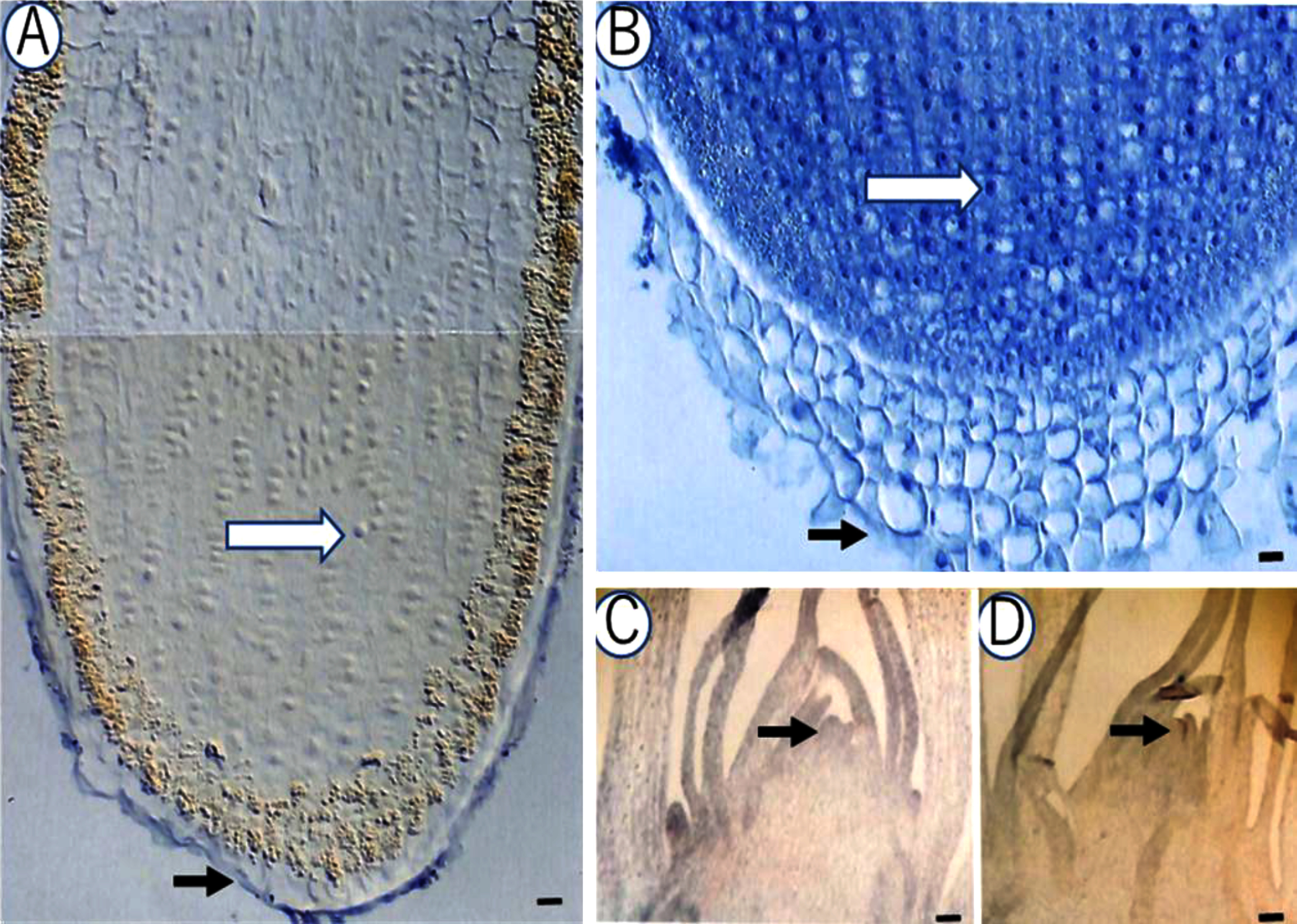
Figure 6. In situ hybridization of *ASG-1* RNA in the longitudinal section of developing vegetative organs from sexual and facultative apomictic *Panicum maximum* plants. (A) Root in the sexual plant showing the meristem (black arrow) and nucleus of cells without signal expression (white arrow). (B) Root in facultative apomictic plants showing the meristem (black arrow) and nucleus of cells with signal expression (white arrow). (C) Shoot apical meristems of a sexual isolate showing no signal, as indicated with the black arrow. (D) Shoot apical meristems of a facultative isolate showing expression, as indicated with the black arrow (Bars in A–B=50 µm; C–D=100 µm).

Because the two genes *ASG-2* and *ASG-1* showed some homology to genes unrelated to reproduction, of auxin downregulated gene [*ADR6p*] (Datta et al. 1993) and polygalacturonase 1 beta chain precursor [*Polyg 1*] (Zheng et al. 1992) or to seed- or embryo-specific genes of other species (Table 2), we further investigated and compared the vegetative tissues of sexual and aposporous *P. maximum* isolates using *ASG-2*. In growing roots, the aposporous apomict showed greater *ASG*-*2* expression in the cell nuclei than in the cytoplasm (Figure 6B). By contrast, no *ASG-2* expression was revealed in any tissues of the sexual isolate (Figure 6A). In the growing shoot apical meristem, *ASG-2* expression was absent in the sexual isolate (Figure 6C) but present in the aposporous apomict (Figure 6D).

## Discussion

For the choice of plant material, we have used a facultative aposporous accession of N68/96-8-o-11 (94% degree of apomixis) and an obligate sexual one of N68/96-8 as the control. These same two materials had also been used in isolation of *ASG-1* (Chen et al. 1999). The materials used in this study were derived from the following process. When the sexual accession of N68/96-8 (2n=4x=32) was open pollinated, 12 plants of the offspring were obtained with degrees of apomixis ranging from 0% to 100% (Nakajima and Mochizuki 1983). Among the plants of the offspring, the aposporous accession of N68/96-8-o-11 (2n=4x=32) observed with modified DIC showed a 94% degree of apomixis (Chen and Kozono 1994a). However, the same accession adapted to the progeny test showed a 100% degree of apomixis, as reported by Nakajima and Mochizuki (1983). And addition, our previous article (Chen and Kozono 1994a) had reported that N68/96-8/o-11 contained 2.5 (1–6) mean numbers of embryo sacs in an ovule, indicating that single aposporous ES has been normally observed in the accession. Expression (degree) of facultativeness was also fluctuated slightly with change in environmental conditions, as when we had used and observed the same facultative accession in different seasons, the different degree of apomixis obtained was changed somewhat (0–<5%) and the data was not published. Thus, based on the two accessions with a close relationship between sisters or mother and child, N68/96-8 was used as the control, and N68/96-8-o-11 was used as the materials for aposporous gene cloning of *ASG-1* (Chen et al. 1999). As the purpose of this study is further to seek any new genes different from the *ASG-1*, an apomixis-specific gene isolated from the facultative aposporous accession of N68/96-8/o-11 of guinea grass (Chen et al. 1999), and analyzed by using in situ hybridization (Chen et al. 2005), that is why we have used the same facultatively aposporous N68/96-8/o-11 in this study.

Using differential screening methods based on the index of the ovary length, four new genes (*ASG-2*, *ASG-3*, *ASG-4*, and *ASG-5*) were cloned from the AIC-stage ovaries of aposporous apomicts of guinea grass N68/96-8-o-11. Our results showed that these transcripts have the same sequences as *ASG-1* but are of different lengths; thus, since *ASG-2* to *5* are clones of varying lengths from the same gene *ASG-1* sharing same start codon, they are considered as the different cDNA clones same to *ASG-1*, designated here as Apomixis-specific gene 1 homologs (*ASG-1H*).

The discovery of the *ASG-1H* has several implications: 1) these genes are expressed and involved specifically in the AIC stage, and they may share some of the same functions and/or may supplement each other; 2) although they were isolated separately, they have the same sequences with different lengths, indicating that they originate from the same region of the genomic DNA; when this region is translated into protein, their expression patterns and differing lengths may be essential and important for AIC appearance; 3) reverse transcription of the isolated RNAs yielded cDNA fragments of different lengths, indicating that they came from the same genomic region because their start sites are the same as those from Poly(A)^+^. Thus, the size differences must result from differences that occur after the start of translation. *ASG-1* expression was detected in AIC and AIC-derived embryo sacs of N68/96-8-o-11 using *ASG-1* as a probe (Chen et al. 2005). Thus, the *ASG-1H* members, with the same sequences as those isolated from AIC-stage ovaries, should have the same functions regarding AIC appearance and their latter actions (Table 3, Figure 4). Our future studies were aimed 1) to use the *ASG-1H* members as probes and retrieve their full genomic sequences to determine whether they come from the same genomic region and 2) to obtain the *ASG-1H* promoter.

Chen et al. (1999) first reported the isolation and characterization of the *ASG-1* gene in aposporous guinea grass. Our literature search of the *ASG-1H* ten years later showed that no further homologies have been described other than RD22 and USP. Our study provides important information indicating that the *ASG-1H* is a new gene and that this gene may be involved not only in AIC and AIC-derived aposporous ES formation but also in further developed ES where the 2n egg cell automatically develops into an embryo with the stimulus of the division of the events in which 2n polar and 1n sperm fuse, divide and form endosperm.

In situ hybridization of ASG-2 was performed comparing aposporous and sexual ES at the AIC stage, indicating that the signals were detected in AIC appearance and AIC-derived ES in the N68/96-8-o-11 aposporous apomict, but no signal was detected in S1 or S2 ES in sexual N68/96-8 (Table 3). This result is the same as that observed for *ASG-1* in a previous report (Chen et al. 2005), indicating that in situ expression analysis can detect signals without distinguishing between *ASG-1* mRNA and other clones of *ASG-1H* in aposporous accession.

At the stages after anthesis, in situ hybridization of ASG-2 has yielded interesting results (Figure 5). At 4 DAA, the first sac in the micropylar end (Figure 5B) had developed both an embryo and endosperm; however, the other sac in the chalazal end contained an 8- to 16-cell embryo and undivided polar nuclei. This result agreed with the first ES appearing always dominantly in the micropylar end and having the temporal dominance in the formation and maturity of the ES; thus, it has the positional dominance in fertilization compared with the other ESs in DIC (Chen and Kozono 1994b) and TEM (Naumova and Willemse 1995). The other sac has a lower chance of fertilization than the first ES (Chen and Kozono 1994b). However, the ES with an 8- to 16-cell embryo was seen along with undivided polar nuclei. This phenomenon can be considered as the stimulus of activity of endosperm formation after fertilization observed not only in *P. maximum* but also in *Paspalum notatum* Flugge L. (Chen, Guan et al. 2001). Naturally, one ovule contains one ES in sexual accession. However, one ovule contains different ESs ranging from one to eight in aposporous *P. maximum* Jacq., naturally, as reported by Chen and Kozono (1994a, b). When comparing the development of embryo/endosperm in different ESs, it was found that the greater the number of ESs, the later the development will be in aposporous accession, and there is no difference of development in single ES’s ovule between sexual and aposporous accessions (Chen and Kozono 1994b). That the development of embryo/endosperm seems faster in sexual accession (Figure 5C, D) than in aposporous accession (Figure 5A, B), as shown in the in situ hybridization of *ASG-2*, is likely due to their different numbers of ESs affecting the speed of development instead of being related to sexual or aposporous accession.

The expression patterns of *ASG-2* in in situ hybridization obtained in this study are the same as that of *ASG-1* during AIC appearance, AIC-derived embryo sac formation, and embryo and endosperm formation (Chen et al. 2005). These data are also supported by RNA gel blot hybridization (Chen et al. 1999). These results suggest that *ASG-2* belongs to the same *ASG-1H* because they were isolated from the same aposporous accession of N68/96-8-o-11.

In other words, multiple mRNAs (cDNAs) of different lengths but identical sequence could be the outcome of alternative splicing of transcripts of the same genes or the expression of duplicated genes due to polyploidy. The Southern blot patterns of A2-512 (*ASG-3*) cDNA (Figure 3), when it was used as the probe and hybridized with genomic DNA purified from leaf tissues, revealed the presence of this gene in both sexual and aposporous isolates of guinea grass, and in which the number and sizes of bands detected in sexual plants were higher than those in aposporous accessions. From the results, the following possibilities could be considered; 1) the locus region of the A2-512 cDNA clone differs largely between the sexual and aposporous accessions; 2) the greater number of bands in the sexual accession means that the A2-512 major gene (gene family) may be multiple alleles in relation to only one band (one allele) of the aposporous accessions; 3) if the upper band of the sexual accessions is removed, the other bands showed similar patterns to those of the aposporous accessions, indicating that apospory may be variants of sexual accessions; 4) only one major band was detected in the aposporous accessions treated with four enzymes, indicating that A2-512 cDNA exists in the locus of the genome and responds to the exon region; 5) by contrast, the multiple bands of sexual accessions treated with the same four enzymes indicate that the exon region may be cut off or a variation may have occurred in the sexual accessions, so no response (bands) were detected in Northern blot hybridization. To sum up the above, when comparing with the A2-512 gene region, the exon region of sexual one may be cut off or a variation may have occurred; therefore, the aposporous phenomenon could not have appeared in the sexual accessions. If the sexual accession N68/96-8 was heterotetraploid, its alleles may be brought in by crossing with another phenotype.

We observed that *ASG-2* is expressed in the meristems of roots and stems of aposporous but not in those of sexual isolates. This finding may be related to the observed homology between *ASG-1* and *ADR6p*, an auxin downregulated gene of the soybean (Datta et al. 1993). The homology to *ADR6p* suggests that *ASG-1GF* members are involved in auxin regulation in tissues undergoing active cell division, such as the tips of roots and meristems of stems, resulting in the observed *ASG-2* expression in these tissues of the apospory (Table 3). The *Polyg 1* gene in the tomato fruit is a probable representative of a class of bifunctional plant proteins that interact with both structural components of the cell wall and catalytic proteins to localize and/or regulate metabolic activities within the cell wall (Zheng et al. 1992). Combining these findings, we suggest that *ASG-2* (*ASG-1GF*), while promoting meristem growth in shoots and roots, may also be involved in auxin regulation and cell wall growth.

In conclusion, the isolation and identification of four new AIC stage-specific cDNAs in aposporous guinea grass carried out in this study suggest that these genes are *ASG-1H*. *ASG-1H* is particularly involved in AIC appearance and AIC-derived aposporous embryo sac formation. As when megaspore mother cells form mature megaspores, AICs, derived from nucellar tissue, appear in the aposporous development of N68/96-8-o-11 (Chen and Kozono 1994a, b), indicating that AIC appearance marks a distinct difference between the two developmental pathways of sexual and apomixis. So we suspect the AIC appearance may be the “turning of the switch” (Chen et al. 1999). Furthermore, combining the results of *ASG-1* and *ASG-1H* that are active in AIC-development and /or the containing ovaries in this study, along with a previous study (Chen et al. 2005), the cloning of the *ASG-1H* expressed at the time of AIC appearance may represent an initial step towards isolating an apospory gene.

In recent years, to establish plant regeneration system for construction of transformation system of the *ASG-1* gene, we have done the establishment of simple plant regeneration system using seeds of apomictic and sexual guinea grass (Chen et al. 2015), and isolation and manipulation of single protoplasts containing aposporous embryo sac initial cell in guinea grass (*Panicum maximum*) (Chen et al. 2014). Next, for applications of various transformation systems on production of *ASG-1* transgenic plants, we have been conducting the establishments of a direct gene transformation system to sweet potato seeds by using electroporation method (Chen et al. 2017), *Agrobacterium*-meditated method transformation system in sweet potato (*Ipomoea batatas*) by using culture of leaf segments (Chen, Nishimura et al. 2013), *Agrobacterium*-mediated transformation system in *Arabidopsis thaliana* (Chen, Xu et al. 2013). In addition, for experimentally functional analysis of *ASG-1* using *Agrobacterium*-mediated method, the following experiment has already been successfully conducted on plant regeneration and functional analysis of *ASG-1* transgenic rice using pSMA35H2-NG (Chen et al. 2016). Together based on several papers on grass plant regeneration published in the Journal Plant Biotechnology, for examples, cultures of leaflets of bahiagrass (Chen, Anami et al. 2001a), embryogenic callus suspension of leaflets bahiagrass (Chen, Anami et al. 2001b), leaflets of guinea grass (Chen et al. 2002a), and ovary of guinea grass (Chen et al. 2002b), we plan to use these culture systems to genetically modify *ASG-1* gene into sexual guinea grass strain and rice. Therefore, this project is expected, like a tiger’s wings to dramatically advance the elucidation of aposporous apomixis.

## References

[RAkiyama2005] Akiyama Y, Hanna WW, Ozias-Akins P (2005) High-resolution physical mapping reveals that the apospory-specific genomic region (ASGR) in *Cenchrus ciliaris* is located on a heterochromatic and hemizygous region of a single chromosome. *Theor Appl Genet* 111: 1042–1051 16133318 10.1007/s00122-005-0020-5

[RAkiyama2008] Akiyama Y, Yamada-Akiyama H, Yamanouchi H, Takahara M, Ebina M, Takamizo T, Sugita S, Nakagawa H (2008) Estimation of genome size and physical mapping of ribosomal DNA in diploid and tetraploid guineagrass (*Panicum maximum* Jacq.). *Grassl Sci* 54: 89–97

[RAltschul1990] Altschul SF, Gish W, Miller W, Myers EW, Lipman DJ (1990) Basic local alignment search tool. *J Mol Biol* 215: 403–410 2231712 10.1016/S0022-2836(05)80360-2

[RAsker1979] Asker SE (1979) Progress in apomixis research. *Hereditas* 91: 231–240

[RAsker1992] Asker SE, Jerling L (1992) Apomixis in plants. *CRC press, Boca Raton*

[RBarcaccia2013] Barcaccia G, Albertini E (2013) Apomixis in plant reproduction: A novel perspective on an old dilemma. *Plant Reprod* 26: 159–179 23852378 10.1007/s00497-013-0222-y PMC3747320

[d69e1704] Bäumlein H, Boerjan W, Nagy I, Bassüner R, Van Montagu M, Inze D, Wobus U (1991) A novel seed protein gene from *Vicia faba* is developmentally regulated in transgenic tobacco and *Arabidopsis* plants. *Mol Gen Genet* 225: 459–467 2017140 10.1007/BF00261688

[RChen2001a] Chen LZ, Anami E, Guan LM, Adachi T (2001a) Somatic embryogenesis and plant regeneration from leaflets of “Nanou” bahiagrass. *Plant Biotechnol (Tokyo)* 18: 119–123

[RChen2001b] Chen LZ, Anami E, Guan LM, Adachi T (2001b) Establishment of embryogenic suspension culture derived from leaflets of sexual bahiagrass (*Paspalum notatum*) with regeneration ability in long term. *Plant Biotechnol (Tokyo)* 18: 209–214

[RChen2000] Chen LZ, Guan LM, Kojima A, Adachi T (2000) The mechanism of appearance of aposporous initial cell and apomictic embryo sac formation in *Paspalum notatum.* *Cytologia (Tokyo)* 65: 333–341

[RChen2001] Chen LZ, Guan LM, Kojima A, Adachi T (2001) The mechanism of polyembryonic seed set in *Paspalum notatum.* *Cytologia (Tokyo)* 66: 157–165

[RChen2005] Chen LZ, Guan LM, Seo M, Hoffmann F, Adachi T (2005) Developmental expression of ASG-1 during gametogenesis in apomictic guinea grass (*Panicum maximum*). *J Plant Physiol* 162: 1141–1148 16255172 10.1016/j.jplph.2005.02.010

[RChen2016] Chen LZ, Guan LM, Toyomoto D, Sugita T, Hamaguchi T, Okabe R (2016) Plant regeneration and its functional analysis within transgenic rice of *ASG-1*, an apomixis specific gene isolated from apomictic guinea grass. *Biotechnol J Int* 16: 1–13

[RChen1994a] Chen LZ, Kozono T (1994a) Cytology and quantitative analysis of aposporous embryo sac development in guinea grass (*Panicum maximum* Jacq.). *Cytologia (Tokyo)* 59: 253–260

[RChen1994b] Chen LZ, Kozono T (1994b) Cytological evidence of seed-forming embryo development in polyembryonic ovules of facultatively apomictic guinea grass (*Panicum maximum* Jacq.). *Cytologia (Tokyo)* 59: 351–359

[RChen2017] Chen LZ, Masuoka S, Nishimura Y, Sakai T, Takahata Y, Xu C (2017) Attempt to establish direct gene transformation system to seeds of sweet potato (*Ipomoea batatas*) using electroporation method. *Biotechnol J Int* 19: 1–8

[RChen1999] Chen LZ, Miyazaki C, Kojima A, Saito A, Adachi T (1999) Isolation and characterization of a gene expressed during early embryo sac development in apomictic guinea grass (*Panicum maximum*). *J Plant Physiol* 154: 55–62

[RChen2013] Chen LZ, Nishimura Y, Tetsumura T, Hamaguchi T, Sugita T, Yoshida K, Xu C (2013) Establishment of *Agrobacterium*-mediated transformation system in *Arabidopsis thaliana* for functional analysis of *ASG*-1, an apomixis specific gene. *Int J Curr Biotech* 1: 6–12

[RChen2015] Chen LZ, Nishimura Y, Umeki K, Zhang J, Xu C (2015) Establishment of a simple plant regeneration system using callus from apomictic and sexual seeds of guinea grass (*Panicum maximum*). *Br Biotechnol J* 7: 183–190

[RChen2014] Chen LZ, Nishimura Y, Xu C (2014) Establishment of efficient system for isolation and manipulation of single protoplasts containing aposporous embryo sac initial cell in guinea grass (*Panicum maximum*). *Br Biotechnol J* 4: 980–989

[RChen2002a] Chen LZ, Okabe R, Guan LM, Adachi T (2002a) A simple and efficient culture of leaflets for plant regeneration in guineagrass (*Panicum maximum*). *Plant Biotechnol (Tokyo)* 19: 63–68

[RChen2002b] Chen LZ, Okabe R, Hamaguchi T, Guan LM, Adachi T (2002b) Effect of harvest seasons on the efficiency of ovary culture in *Panicum maximum.* *Plant Biotechnol (Tokyo)* 19: 173–179

[RChen2013b] Chen LZ, Xu C, Du ZS, Hamaguchi T, Sugita T, Ichikawa H, Guan LM (2013) Establishment of *Agrobacterium*-meditated method transformation system in sweet potato (*Ipomoea batatas*) by culture of leaf segments for functional analysis of *ASG-1*, an apomixis-specific gene. *Br Biotechnol J* 3: 458–470

[RConner2015] Conner JA, Mookkan M, Huo H, Chae K, Ozias-Akins P (2015) A parthenogenesis gene of apomict origin elicit embryo formation from unfertilized eggs in a sexual plant. *Proc Natl Acad Sci USA* 112: 11205–11210 26305939 10.1073/pnas.1505856112 PMC4568661

[RDatta1993] Datta N, LaFayette PR, Kroner PA, Nagao RT, Key JL (1993) Isolation and characterization of three families of auxin down-regulated cDNA clones. *Plant Mol Biol* 21: 859–869 8096772 10.1007/BF00027117

[REbina2005] Ebina M, Nakagawa H, Yamamoto T, Araya H, Tsurata S, Takahara M, Nakajima K (2005) Co-segregation of AFLP and RAPD markers to apospory in guineagrass (*Panicum maximum* Jacq.). *Grassl Sci* 51: 71–78

[RGuan2006] Guan LM, Chen LZ, Terao H (2006) Ultrastructural studies of gametophytic apomicts in guinea grass (*Panicum maximum*). I. Differentiation of aposporous initial cell. *Cytologia (Tokyo)* 71: 379–389

[RGuan2007] Guan LM, Chen LZ, Terao H (2007) Ultrastructural studies of Gametophytic Apomicts in Guinea grass (*Panicum maximum*) II. Characteristics of aposporous initial cell-derived embryo sac. *Cytologia (Tokyo)* 72: 145–153

[RGuerin2000] Guerin J, Rossel JB, Robert S, Tsuchiya T, Koltunow AM (2000) *DEFICIENS* homologue is down-regulated during apomictic initiation in ovules of *Hieracium.* *Planta* 210: 914–920 10872222 10.1007/s004250050697

[RHand2014] Hand ML, Koltunow AM (2014) The genetic control of apomixis: Asexual seed formation. *Genetics* 197: 441–450 24939990 10.1534/genetics.114.163105 PMC4063905

[RHanna1973] Hanna WW, Powell J, Millot JC, Burton GW (1973) Cytology of obligate sexual plants in *Panicum maximum* Jacq. and their use in controlled hybrids. *Crop Sci* 13: 695–697

[RHonda1990] Honda H, Hirai A (1990) A simple and efficient method for identification of hybrids using nonradioactive rDNA as probe. *Japan J Breed* 40: 339–348

[RKhanday2019] Khanday I, Skinner D, Yang B, Mercier R, Sundaresan V (2019) A male-expressed rice embryogenic trigger redirected for asexual propagation through seeds. *Nature* 565: 91–95 30542157 10.1038/s41586-018-0785-8

[RKoltunow1993] Koltunow AM (1993) Apomixis: embryo sac and embryos formed without meiosis or fertilization in ovules. *Plant Cell* 5: 1425–1437 12271038 10.1105/tpc.5.10.1425 PMC160373

[RKoltunow1995] Koltunow AM, Bicknell RA, Chaudhury AM (1995) Apomixis: Molecular strategies for the generation of genetically identical seeds without fertilization. *Plant Physiol* 108: 1345–1352 12228546 10.1104/pp.108.4.1345 PMC157511

[d69e2326] León-Martínez G, Vielle-Calzada JP (2019) Apomixis in flowering plants: developmental and evolutionary considerations. *Curr Top Dev Biol* 131: 565–604 30612631 10.1016/bs.ctdb.2018.11.014

[RNakagawa1990] Nakagawa H (1990) Embryo sac analysis and crossing procedure for breeding apomictic guineagrass (*Panicum maximum* Jacq.). *Jpn Agric Res Q* 24: 163–168

[RNakajima1983] Nakajima K, Mochizuki N (1983) Degrees of sexuality in sexual plants of guineagrass by the simplified embryo sac analysis. *Japan J Breed* 33: 45–54

[RNaumova1995] Naumova TN, Willemse MTM (1995) Ultrastructural characterization of apospory in *Panicum maximum.* *Sex Plant Reprod* 8: 197–204

[RNogler1984] Nogler GA (1984) Gametophytic apomixis. In: Johri BM (ed) *Embryology of Angiosperm. Springer-Verlag Berlin*, pp 475–518

[ROzias-Akins1998] Ozias-Akins P, Roche D, Hanna WW (1998) Tight clustering and hemizygosity of apomixis-linked molecular markers in *Pennisetum squamulatum* implies genetic control of apospory by a divergent locus that may have no allelic form in sexual genotypes. *Proc Natl Acad Sci USA* 95: 5127–5132 9560240 10.1073/pnas.95.9.5127 PMC20225

[RRavi2008] Ravi M, Marimuthu MPA, Siddiqi I (2008) Gamete formation without meiosis in *Arabidopsis.* *Nature* 451: 1121–1124 18272967 10.1038/nature06557

[RSavidan1975] Savidan Y (1975) Heredite de l’apomixie, contribution a l’etude de l’heredite de l’apomixie sue *panicum maximum* Jacq. (analyse des sacs embryonnaires). *Cah ORSTOM Ser Biol* 10: 91–95

[RSavidan1982] Savidan Y, Pernès J (1982) Diploid-tetraploid cycles and the evolution of *Panicum maximum* Jacq. *Evolution* 36: 596–600 28568051 10.1111/j.1558-5646.1982.tb05081.x

[RSmith1972] Smith RL (1972) Sexual reproduction in *Panicum maximum* Jacq. *Crop Sci* 12: 624–627

[RSouthern1975] Southern EM (1975) Detection of specific sequences among DNA fragments separated by gel electrophoresis. *J Mol Biol* 98: 503–517 1195397 10.1016/s0022-2836(75)80083-0

[RUnderwood2022] Underwood CJ, Vijverberg K, Rigola D, Okamoto S, Oplaat C, Camp RHMOD, Radoeva T, Schauer SE, Fierens J, Jansen K, et al. (2022) A *PARTHENOGENESIS* allele from apomictic dandelion can induce egg cell division without fertilization in lettuce. *Nat Genet* 54: 84–93 34992267 10.1038/s41588-021-00984-y

[RVernet2022] Vernet A, Meynard D, Lian Q, Mieulet D, Gibert O, Bissah M, Rivallan R, Autran D, Leblanc O, Meunier AC, et al. (2022) High-frequency synthetic apomixis in hybrid rice. *Nat Commun* 13: 7963 36575169 10.1038/s41467-022-35679-3 PMC9794695

[RWarmke1954] Warmke HE (1954) Apomixis in *Panicum maximum.* *Am J Bot* 41: 5–11

[RYamaguchi-Shinozaki1993] Yamaguchi-Shinozaki K, Shinozaki K (1993) The plant hormone abscisic acid mediates the drought-induced expression but not the seed-specific expression of rd22, a gene responsive to dehydration stress in *Arabidopsis thaliana.* *Mol Gen Genet* 238: 17–25 8479424 10.1007/BF00279525

[RZheng1992] Zheng L, Heupel RC, DellaPenna D (1992) The beta subunit of tomato fruit polygalacturonase isoenzyme 1: isolation, characterization, and identification of unique structural features. *Plant Cell* 4: 1147–1156 1392611 10.1105/tpc.4.9.1147 PMC160205

